# Is It Time We Stop Discouraging Evening Physical Activity? New Real-World Evidence From 150,000 Nights

**DOI:** 10.3389/fpubh.2021.772376

**Published:** 2021-11-04

**Authors:** Michal Kahn, Topi Korhonen, Leena Leinonen, Kaisu Martinmaki, Liisa Kuula, Anu-Katriina Pesonen, Michael Gradisar

**Affiliations:** ^1^College of Education, Psychology and Social Work, Flinders University, Adelaide, SA, Australia; ^2^Polar Electro Oy, Polar Research Center, Kempele, Finland; ^3^SleepWell Research Program, Faculty of Medicine, University of Helsinki, Helsinki, Finland

**Keywords:** physical activity, evening exercise, sleep hygiene, sleep quality, sleep duration, big-data

## Abstract

Professional and colloquial sleep hygiene guidelines advise against evening physical activity, despite meta-analyses of laboratory studies concluding that evening exercise does not impair sleep. This study is the first to investigate the association between objectively measured evening physical activity and sleep within a real-world big-data sample. A total of 153,154 nights from 12,638 individuals aged 18–60 years (*M* = 40.1 *SD* = 10.1; 44.5% female) were analyzed. Nighttime sleep and minutes of physical activity were assessed using Polar wearable devices for 14 consecutive days. Thirty minutes or more of moderate-to-near maximal physical activity during the 3 h before sleep onset were recorded in 12.4% of evenings, and were more frequent on weekdays than weekends (13.3 vs. 10.2% respectively, *p* < 0.001). Linear mixed modeling revealed that sleep efficiency was not significantly associated with evening physical activity, and that sleep duration was 3.4 min longer on average on nights following evenings in which participants engaged in 30 min or more of moderate-intense physical activity. Effects were found for sleep timing metrics, as evening physical activity was linked with earlier sleep onset and offset times (−13.7 and −9.3 min, respectively). Overall, these effects were greater– but still very small– on weekdays compared to weekends. The present study provides further evidence for the lack of meaningful links between sleep duration or quality and physical activity in the hours preceding sleep. Taken together with recent meta-analytic findings, these findings suggest that changes in public health recommendations are warranted regarding evening physical activity and its relation to sleep.

## Introduction

Sleep and exercise are both imperative for physical and mental health (1–3). Yet, to promote healthy sleep, long-standing guidelines have advised against evening exercise. For example, the American Sleep Association's “*Sleep Hygiene Tips*” include exercising before 2 P.M., and avoiding rigorous exercise before bedtime (4). Such recommendations have been based on the premise that the elevation of core body temperature and increase in physiological arousal resulting from evening physical activity (PA), can delay the circadian rhythm and disrupt sleep (5, 6). However, in modern society, allocating time for exercise early in the day may be highly challenging. In a nationally representative survey of 15,239 Europeans, the most commonly endorsed barrier to increase PA was work or study commitments (7). Hence, discouraging evening PA may come at the high cost of reducing overall PA for individuals who otherwise would have engaged in exercise later in the day.

Research to date has yielded mixed findings with regards to the effects of evening PA on sleep. Several studies have reported increased sleep onset latency (SOL) following evening exercise [e.g., (8)], whilst other studies found null [e.g., (9)] or positive [e.g., (10)] effects on sleep. A meta-analysis synthesizing data from 23 experimental studies of evening PA on sleep found no difference in SOL, sleep efficiency (SE), total sleep time (TST), or subjective sleep quality when compared to no-exercise (11). Despite some indication for increased SOL following intense PA performed very close to bedtime, the authors concluded that evening exercise does not impair sleep (11). These conclusions are consistent with a slightly earlier meta-analysis, which compared the effects of acute moderate-to-vigorous PA performed >8, 3–8, and <3 h before bedtime, revealing no differences in SOL, SE, or TST as a function of PA timing (12).

Complimentary to experimental studies, epidemiologic studies can provide large-scale, ecologically valid information about the links between evening PA and sleep. In the largest survey study to date, Buman et al. (13) examined these links in 1000 US adults, and found no differences in reported TST, SOL, sleep quality or the experience of waking unrefreshed between individuals that habitually engage in moderate or vigorous exercise in the evenings and non-exercisers. Importantly, 23% of participants engaged in moderate or vigorous evening PA, and most of those believed that their sleep was of equal or better quality (97%) and of equal or longer duration (98%) *following* evening exercise. This investigation provided further evidence for the need to reconsider guidelines discouraging evening PA. However, it was based on retrospective reporting of both exercise and sleep, and thus subject to social and recall biases (14).

The increasing prevalence of commercially-available wearable devices allows for exploration of naturalistic *objective* sleep and PA measures in big-data samples (15, 16). To the best of our knowledge, thus far only one study has used big-data to evaluate the links between sleep and PA performed during the day, yet the links between sleep and evening PA were not assessed (17). Therefore, the main aim of the present study was to evaluate the associations between accelerometer-measured sleep and moderate to intense PA in the 3 h before bedtime in a real-world big-data sample.

A further aim of the present study was testing whether the association between sleep and evening PA is moderated by their timing within the week. Previous investigations have examined the moderating effects of various factors, including the type, duration, and intensity of exercise (11, 12). Whether the association of evening PA and sleep differs on weekdays vs. weekends, however, has yet to be explored. Being strongly affected by social factors and daily schedules, both sleep and PA patterns may vary as a function of their timing within the week. Previous work has demonstrated delayed sleep timing and increased sleep duration on free days compared to workdays (15, 18–20). PA patterns have also been shown to vary throughout the week, with some individuals demonstrating a “weekend warrior” pattern, in which most or all of weekly PA is performed on weekends (21). A recent study exploring time use patterns of young adults in the 3 h before bed revealed vast individual differences in the type and duration of activities endorsed, but did not examine whether these activities differ on weekdays vs. weekends (22). Using objective assessment of PA and sleep patterns across multiple days, the present study aimed to evaluate whether evening PA patterns were equivalent on weekdays compared to weekdays, and whether the association between evening PA and sleep metrics varied between weekdays and weekends.

## Materials and Methods

### Participants and Procedures

Data from a total of 12,638 individuals aged 18–60 years (*M* = 40.1 *SD* = 10.1; 44.5% female) were included in this study. Data were obtained between January 2018 and December 2018. When taking their Polar watches into use, users agreed that their Polar data could be used for Polar research and development purposes. Polar researchers handled the data anonymously and performed the statistical analysis for this study inside the company. Randomly selected individuals were included in the sample if they had two consecutive weeks of available sleep and physical activity data, as well as complete demographic data, including age, gender, height, weight, and country of residence. Nights of sleep assessment were considered available if the duration of time from sleep onset to offset was between 4 and 13 h, and the total (true) sleep duration was between 3 and 13 h. Days of physical activity assessment were considered available if the device was worn for ≥10 h of wakefulness per 24-h day. Height and weight were used to compute a BMI score for each participant (Range: 15–45; *M* = 25.7 *SD* = 4.1).

### Measures

Nighttime sleep and physical activity in the 3 h before sleep onset were assessed using Polar devices (models A370, M430, M600, Vantage V, and Vantage M). These wrist-worn devices all use validated proprietary algorithms to automatically translate biosignals into PA and sleep-wake metrics (23, 24). PA tracking fuses acceleration and heart rate data, and sleep-wake detection utilizes acceleration data only. For the purpose of the present study, derived sleep metrics included sleep onset time, sleep offset time, total sleep duration (calculated as the duration of time between sleep onset and offset, excluding wakefulness within that period), and sleep efficiency (calculated as the percent of total sleep duration out of the duration of time between sleep onset and offset).

Physical activity levels were quantified per 30 s epochs and classified into one of the following categories: (1) sedentary or very light physical activity, with Metabolic Equivalent of Task (MET) values <2; (2) light physical activity, with MET values between 2 and 2.95; (3) moderate physical activity, with MET values between 2.95 and 5.95; (4) vigorous physical activity, with MET values between 5.95 and 8.75; or (5) near maximal physical activity, with MET values ≥8.75 (25). Epochs were then summed per PA category across 30-min periods within the 3 h before sleep onset. Finally, mins of moderate, vigorous, and near maximal PA were summed across the 3 h before sleep onset, and a binary measure was created, categorizing nights into either having ≤ 30 or >30 min of moderate to near maximal PA within the 3 h before sleep onset.

### Data Analysis Plan

Data processing was performed in Python (v3.8.5) and R (v4.1.0) using RStudio (v1.4.1717). Outlier sleep values (± 3SD from the mean) were excluded, as were evening PA values in which the device was not worn for the entire 3 h before sleep onset, and there were ≤ 30 min of moderate to near maximal PA within that period. This resulted in exclusion of 13.4% of individual evenings/nights, bringing the average number of evenings/nights per participant to 12.1, and the total number of analyzed evenings/nights to 153,154.

We computed Pearson correlations between each sleep metric and minutes of moderate-to-near maximal PA in the six 30-min time slots leading up to sleep onset. These were computed separately for weekdays and weekends. Linear mixed models were used to compare sleep after evenings with *over* and *under* 30 min of moderate-to-near maximal PA during the 3 h before bedtime. Mixed modeling considers all available data points, accounts for the nested nature of the data (i.e., multiple evenings/nights nested within participant), and can include random– in addition to fixed– effects. Models were computed using the “lme4” package (26). Model terms included PA (≤ 30 vs. >30 min of moderate to near maximal physical activity within the 3 h before sleep onset), controlling for participant age, gender, BMI, and weekend (vs. weekday). PA-by-weekend interaction terms were then added to each model, to test whether the association between PA and sleep varied between weekdays and weekends. Models including random slopes yielded significantly lower deviance compared to those excluding them, and are thus reported. *P*-values were computed using Satterhwaite's method, obtained via the lmer() “summary” function. Given the large sample size, significance levels were adjusted to 0.004, based on Good's standardization formula (27). Cohen's *d* effect sizes were calculated using the “effsize” package (28). Pairwise comparisons were computed for significant interaction terms using the “emmeans” package (29).

## Results

**Figure 1:**
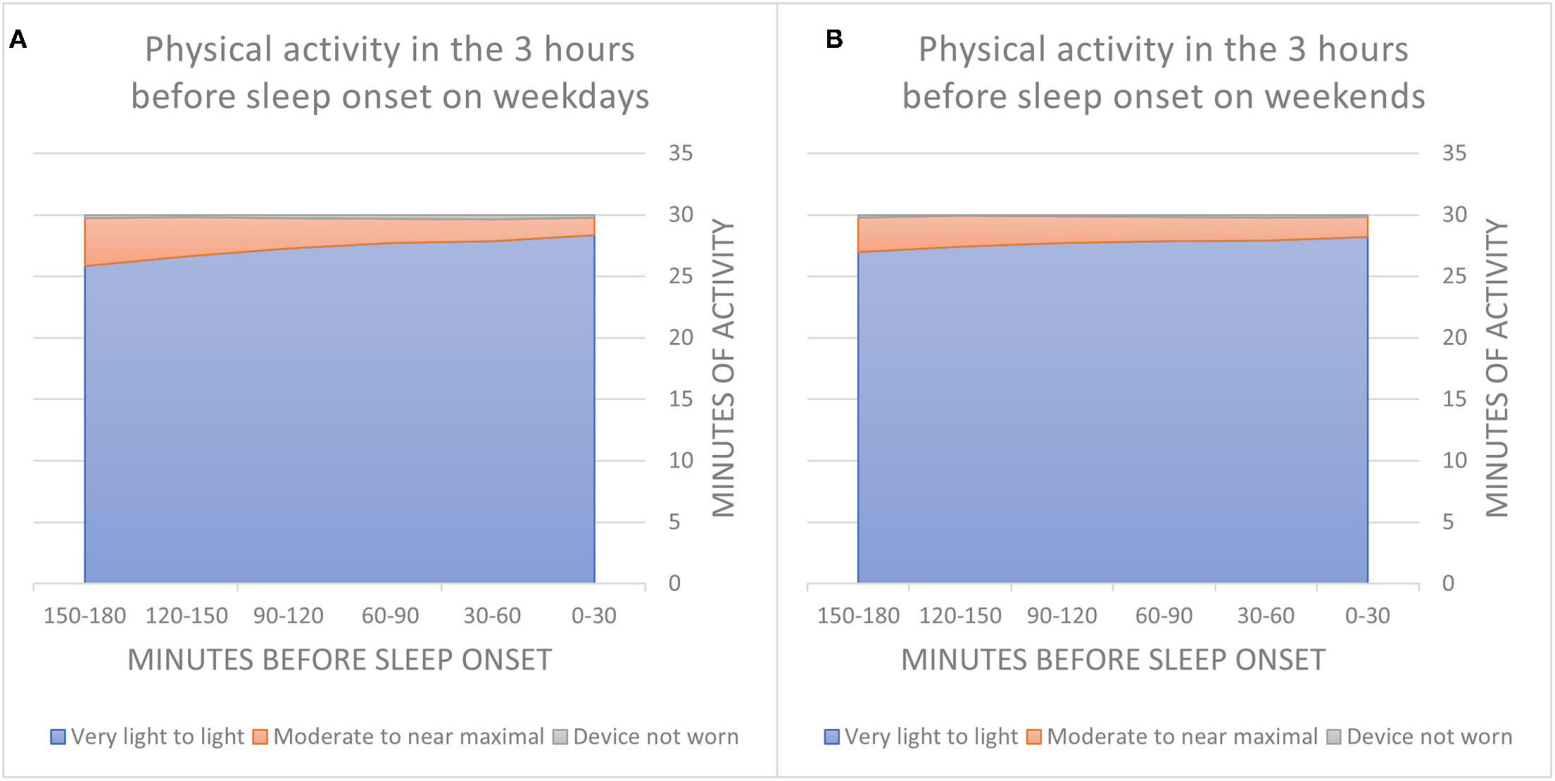
Average minutes of physical activity per 30-min in the 3 h leading up to sleep onset on weekday evenings **(A)** and weekend evenings **(B)**.

Descriptive statistics for PA categories in the 3 h leading up to sleep onset on weekdays and weekends are presented in Figure 1. Participants mostly engaged in sedentary to light activity during these hours, with the average duration of moderate-to-near maximal PA ranging from 1.4 to 3.9 min (*SD* = 0.6–2.1) per 30-min period. Thirty minutes or more of moderate-to-near maximal PA during the 3 h before sleep onset were recorded in 12.4% of evenings. Inspection of weekday and weekend patterns revealed a significant difference, with PA performed on 13.3% and 10.2% of weekday and weekend evenings, respectively [χ^2^(1) = 275.02, *p* < 0.001]. As for the proportion of participants engaging in evening PA, 7425 participants (59%) had at least one evening with >30 min of moderate-to-near maximal PA in the 3 h leading up to sleep onset, 4,531 (35.8%) had ≥2 evenings, 2,781 (22.0%) had ≥3 evenings, 1,721 (13.6%) had ≥4 evenings, and 1,050 (8.3%) had ≥5 such evenings within the 2-week assessment period.

Correlations between sleep metrics and duration of evening moderate-to-intense PA during weekdays and weekends are presented in Table 1. Overall, associations were weak, and ranged between negligible and very small. The latter represented correlations between earlier sleep onset and offset times and more PA in the hour ending 2 h before sleep onset on weeknights. Notably, correlations with sleep timing metrics were greater for PA performed earlier in the evening compared to directly before sleep onset. Finally, stronger correlations were found between evening PA and sleep on weekday compared to weekend evenings/nights. Yet, these correlations were still very weak (maximum *r* = −0.16).

**Table 1:** Zero-order Pearson correlations between weekday and weekend sleep metrics and duration of moderate to near-maximal physical activity in every 30-min period in the 3 h before sleep onset.

		**Minutes of moderate to near maximal physical activity per 30-min period leading up to sleep onset**					
		**180–150 min**	**150–120 min**	**120–90 min**	**90–60 min**	**60–30 min**	**30 min–sleep onset**
Weekdays	Total sleep time	−0.01	−0.01	−0.02	−0.03	−0.04^***^	−0.01
	Sleep Efficiency	0.00	0.00	−0.01	−0.03	0.00	0.07^***^
	Sleep onset	−0.16^***^	−0.16^***^	−0.14^***^	−0.11^***^	−0.07^***^	−0.10^***^
	Sleep offset	−0.16^***^	−0.16^***^	−0.14^***^	−0.12^***^	−0.10^***^	−0.11^***^
Weekends	Total sleep time	−0.03	−0.03^*^	−0.04^***^	−0.04^**^	−0.04^***^	0.00
	Sleep efficiency	−0.04^**^	−0.03	−0.03	−0.03	−0.01	0.05^***^
	Sleep onset	−0.06^***^	−0.05^***^	−0.03	0.01	0.04^***^	−0.01
	Sleep offset	−0.07^***^	−0.07^***^	−0.06^***^	−0.01	0.01	−0.01

*As in a heatmap, stronger correlations are highlighted by darker shades, with blue representing correlations smaller than the mean and red representing correlations greater than the mean*.

* *p < 0.001*,

** *p < 0.0001*,

*** *p < 0.00001*.

**Figure 2:**
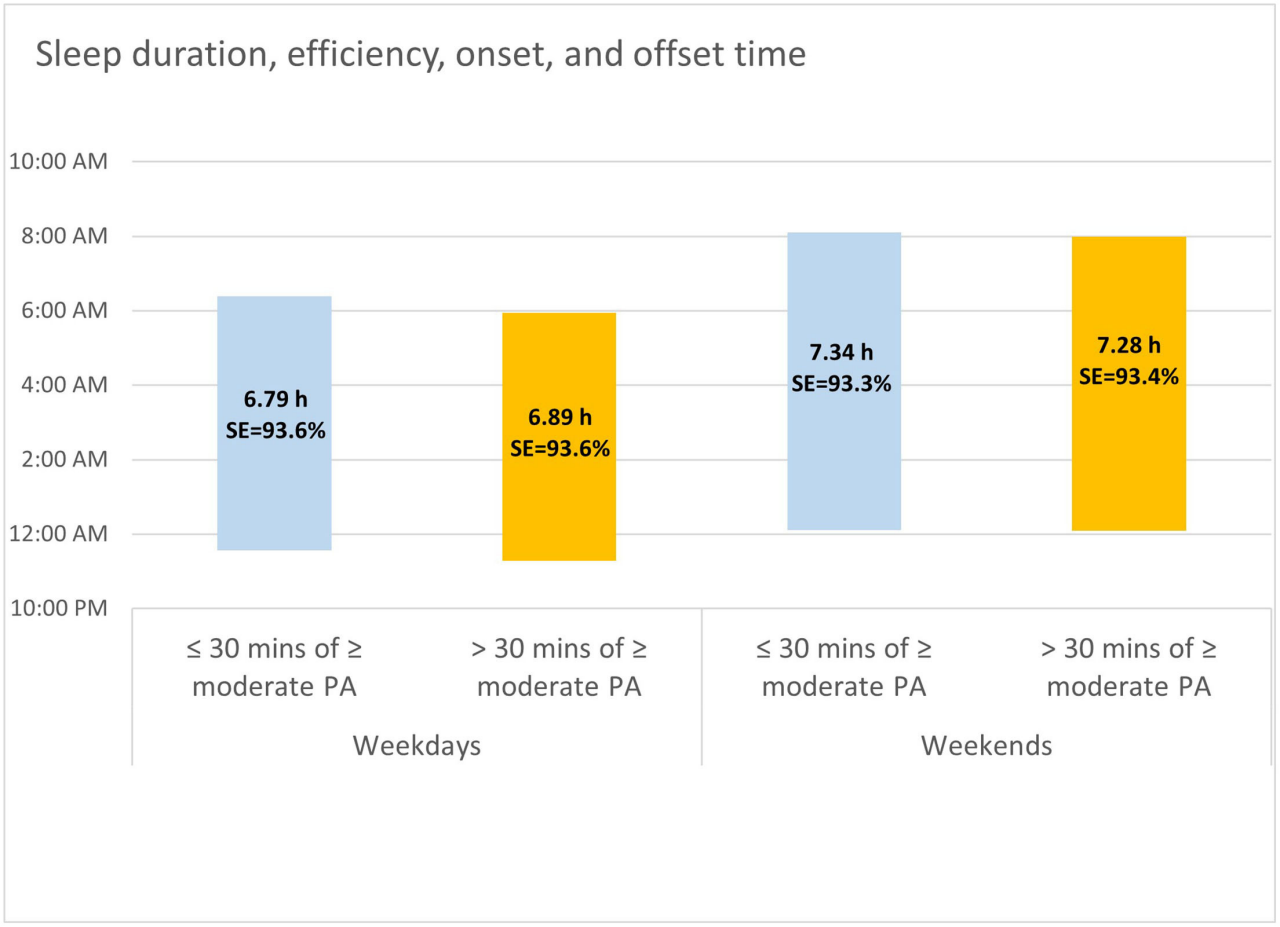
Sleep timing, duration, and efficiency on weekday and weekend nights that were preceded by ≤ 30 vs. >30 min of moderate to near maximal physical activity in the 3 h before sleep onset.

Linear mixed models testing whether sleep differed on nights that were preceded with less compared to more than 30 min of PA were computed for each sleep metric. Models yielded a non-significant main effect of PA for sleep efficiency (*t* = 1.62, *p* = 0.11), indicating that sleep efficiency was not associated with evening PA. Sleep duration, on the other hand, was significantly associated with evening PA (*t* = 6.20, *p* < 0.0001). On nights following evenings in which participants engaged in 30 min or more of moderate-intense PA, sleep duration was 3.4 min longer on average (Cohen's *d* = −0.01, CI 95% = −0.004–0.03). Main effects were also indexed for sleep timing metrics. Sleep onset time was 13.7 min earlier on average (Cohen's *d* = 0.26, CI 95% = 0.24–0.27; *t* = −26.20, *p* < 0.0001), and sleep offset was 9.3 min earlier (Cohen's *d* = 0.25, CI 95% = 0.23–0.26; *t* = −17.19, *p* < 0.0001) when users had 30 min or more of moderate-intense PA in the 3 h before bedtime (see Figure 2).

Whereas the link between PA and sleep efficiency was similar for both weekdays and weekends (*t* = 1.32, *p* = 0.19), models yielded significant PA-by-weekend interaction effects for sleep duration (*t* = −7.98, *p* < 0.0001), sleep onset (*t* = 13.00, *p* < 0.0001), and sleep offset times (*t* = 3.66, *p* = 0.0002). Overall, pairwise comparisons indexed greater differences in sleep as a function of PA on weekdays compared to weekends. Specifically, following > 30 min of moderate to intense PA participants slept for longer durations on weekdays (*M*_*d*__ifference_= 5.7 min, *SE* = 0.6, *p* < 0.0001), and shorter durations on weekends (*M*_*d*__ifference_= −3.8 min, *SE* = 1.0, *p* = 0.0003). Moreover, earlier sleep onset was recorded on weekday nights that were preceded by > 30 min of moderate to intense PA (*M*_*d*__ifference_= 17.3 min, *SE* = 0.6, *p* < 0.0001), but not on weekend nights (*M*_*d*__ifference_= 2.6 min, *SE* = 1.0, *p* = 0.01). Finally, earlier sleep offset was recorded on both weekdays and weekends, yet the difference was greater on weekdays compared to weekends (*M*_*d*__ifference_= 10.3 min, *SE* = 0.6, *p* < 0.0001 vs. *M*_*d*__ifference_= 6.1 min, *SE* = 1.0, *p* < 0.0001; respectively).

## Discussion

Traditional guidelines promoting healthy sleep hygiene have discouraged evening physical activity. Such guidelines have been voiced by professional healthcare organizations [e.g., (4)], and are regularly echoed in colloquial sleep health information and tips proposed in media outlets around the world [e.g., (30)]. Yet, existing evidence does not attest to a reliable association between evening exercise and impaired sleep (11, 12). Utilizing an objective big-data sample, the present study provides further evidence for the lack of meaningful links between sleep duration or quality and PA in the hours preceding sleep.

Our findings revealed negligible-to-weak associations between sleep metrics and moderate-to-near maximal PA within the 3 h leading to sleep onset. When adjusted for potentially confounding variables, evening PA was not significantly linked with sleep efficiency. Adjusted models did yield a significant link between sleep duration and evening PA. However, engaging in over 30 min of moderate to intense PA was followed by a negligible 3.4 min of additional sleep on average. These findings dovetail with the two latest meta-analyses of experimental studies addressing this link. Stutz et al. (11) found that evening exercise yielded non-significant pooled increases for both sleep efficiency (+0.13%) and sleep duration (+1.04 min), compared to a no-exercise control condition. Kredlow et al.'s meta-analysis (12) found that exercise had small beneficial effects on sleep efficiency as well as on total sleep time, regardless of whether it was performed >8, 3–8, or <3 h before bedtime. Epidemiologic studies using self-report measures have added to this literature, in demonstrating a lack of connection between evening exercise and impaired sleep (13, 31). Taken together with the results of the present study, associations between evening PA and sleep quality and quantity seem to range from null to positive, with no indication of evening PA being associated with poorer or shorter sleep.

The minor impact of evening PA on sleep duration may be due to several underlying processes. First, the acute body-heating during PA is typically followed by core temperature downregulation in the subsequent few hours, which may facilitate sleep (32). Second, in contrast to the theoretical notion of evening PA hindering sleep by causing excessive arousal, PA has been shown to have a regulating, anxiolytic effect, particularly in the 1–2 h after exercising (33), which may promote sleep. Finally, evening exercise may strengthen beneficial sleep associations, by contrasting higher activity levels outside the bed with restful inactivity within the bed, in line with the classical conditioning rationale (34). These mechanistic processes may promote longer sleep duration largely by decreasing SOL at the start of the night, thus allowing more time for sleep. Whilst SOL was not assessed in the present study, the lack of significant effect for SE implies that a slight reduction in SOL might indeed have driven the small extension in sleep duration following >30 min of evening PA.

As for sleep timing, our analyses revealed significantly earlier sleep onset (−13.7 min) and offset (−9.3 min) times following nights on which >30 min of moderate-to-near maximal PA was performed in the 3 h before bedtime. The effects of evening exercise on sleep timing have received considerably less research attention relative to sleep duration and quality metrics, and experimental studies have often included prescribed sleep schedules, not allowing for assessment of naturalistic sleep timing following PA. Still, investigations into the phase-shifting potential of evening exercise as an internal zeitgeber offer some insight on the effects that evening PA may have on sleep timing. Several laboratory investigations have indicated circadian phase delays after exercise performed in the hours before bedtime (6, 35, 36). For example, Youngstedt et al. (6) observed phase delays following exercise at 7 and 10 P.M. using multiple circadian markers, in both younger and older women and men. Other studies, however, found that evening PA had no effects (37), or circadian phase-*advancing* effects (38). Given that circadian rhythms were not directly assessed in the present study, it remains unclear whether phase-advancing effects contributed to the earlier sleep timing found following evening PA. Nevertheless, these advances in sleep timing were small, and did not impact TST (e.g., a 13.7-min advanced sleep onset did not equate to a 13.7-min increase in TST).

This study additionally found that links between evening PA and sleep were weaker on weekends compared to weekdays. Professional and educational commitments tend to be more relaxed on weekends, allowing more time for PA throughout the day (21). Accordingly, our results also indexed that engaging in >30 min of moderate to intense PA in the 3 h before bed was less frequent on weekends compared to weekdays.

### Strengths and Limitations

This study had several strengths. It utilized objective assessment of both PA and sleep, thus reducing reporting bias. As these devices are regularly used by individuals for tracking their own sleep and activity in naturalistic settings, they provide real-world ecologically valid data, as opposed to data generated to conform with external requirements, such as participation in research (15). Finally, while the largest sample to date to examine the links between evening PA and sleep used 1,000 participants at a single timepoint, data in the present study were collected from 12,638 participants over a 2-week period, resulting in 153,154 analyzed evenings and nights.

Despite these strengths, several limitations exist. First, this study does not allow for conclusions about directionality or causality. Research and policy in this area have mostly focused on the potential negative effects of evening PA on subsequent nighttime sleep. However, given the bi-directional links between these behaviors (39), nighttime sleep may also impact on next day evening PA. Moreover, given that this was an uncontrolled study, users' natural preferences were not accounted for. It may be, for example, that individuals who have learned that their sleep is impaired if they exercise in the evening– simply refrain from it. Similarly, we could not account for the effects of other individual characteristics, such as fitness levels, chronotypes, sleep disorders, or other medical conditions. Furthermore, minutes of moderate to near maximal PA may have been consecutive or sporadic within the 3 h before sleep onset, and data were not collected regarding the type of activity performed, or the environmental conditions (e.g., light and temperature) in which it was carried out. These factors have been shown to moderate the PA-sleep link in previous studies (11, 40), and thus adjusting for them could have resulted in a more accurate and intricate account of the associations between evening PA and nighttime sleep.

Finally, the generalizability of our findings may be limited by selection bias, as users of wearable fitness tracker devices may more likely engage in health promoting behaviors compared to the general population. Nonetheless, the growing popularity and pervasiveness of such devices suggests that their users do not represent an outlier, but rather a population not far from the norm. In fact, 13.6% of participants in our sample had 4 evenings or more of >30 min PA in the 3 h before bed within the 2-week assessment period. This rate coincides with prevalence of regular evening moderate and vigorous exercisers (10.4 and 13.2%, respectively), found in a representative adult sample in the USA (13).

## Conclusions

While current official and colloquial recommendations suggest that PA before bed impairs sleep (4), the present study demonstrates that moderate to vigorous activity in the 3 h before bed is unrelated to sleep quality, and related to slightly *longer* sleep duration and earlier sleep timing. Given the numerous beneficial effects of PA for health and well-being, and given the challenge of fitting PA earlier in the day for many citizens of modern societies, we argue that public health guidelines should urgently be changed. Moreover, active attempts should be pursued to amend the common misperception that evening PA impairs sleep. While further research is warranted to inform recommendations for specific populations and circumstances, a clear message should be delivered to the general population, stating that evening PA should no longer be avoided.

## Data Availability Statement

The datasets presented in this article are not openly available given the need to preserve the privacy of participating individuals. Requests to access the datasets should be directed to kaisu.martinmaki@polar.com.

## Ethics Statement

Ethical review and approval was not required for the study on human participants in accordance with the local legislation and institutional requirements. The patients/participants provided their written informed consent to participate in this study.

## Author Contributions

MK performed the literature review, planned the data analysis, and drafted the manuscript. TK performed the data analysis and revised the manuscript. LL performed data extraction and revised the manuscript. KM was responsible for project administration and revised the manuscript. LK, A-KP, and MG conceptualized the study and revised the manuscript. All authors substantially contributed to the study and approved the submitted version.

## Funding

This research received funding from Polar to cover the costs of open access publishing. Polar also provided the data and performed the statistical analysis for this study.

## Conflict of Interest

TK, LL, and KM were employees of Polar at the time of data collection and analysis. The remaining authors declare that the research was conducted in the absence of any commercial or financial relationships that could be construed as a potential conflict of interest.

## Publisher's Note

All claims expressed in this article are solely those of the authors and do not necessarily represent those of their affiliated organizations, or those of the publisher, the editors and the reviewers. Any product that may be evaluated in this article, or claim that may be made by its manufacturer, is not guaranteed or endorsed by the publisher.
